# Wireless, battery-free, and fully implantable electrical neurostimulation in freely moving rodents

**DOI:** 10.1038/s41378-021-00294-7

**Published:** 2021-08-13

**Authors:** Alex Burton, Sang Min Won, Arian Kolahi Sohrabi, Tucker Stuart, Amir Amirhossein, Jong Uk Kim, Yoonseok Park, Andrew Gabros, John A. Rogers, Flavia Vitale, Andrew G. Richardson, Philipp Gutruf

**Affiliations:** 1 Department of Biomedical Engineering, University of Arizona, Tucson, AZ 85721 USA; 2 Department of Electrical and Computer Engineering, Sungkyunkwan University (SKKU), Suwon, 16419 Republic of Korea; 3 Department of Neurosurgery, Perelman School of Medicine, University of Pennsylvania, Philadelphia, PA 19104 USA; 4 Querrey Simpson Institute for Bioelectronics, Northwestern University, Evanston, IL 60208 USA; 5 Center for Bio-Integrated Electronics, Northwestern University, Evanston, IL 60208 USA; 6 Department of Mechanical Engineering, Northwestern University, Evanston, IL 60208 USA; 7 Department of Biomedical Engineering, Northwestern University, Evanston, IL 60208 USA; 8 Department of Materials Science and Engineering, Northwestern University, Evanston, IL 60208 USA; 9 Department of Neurological Surgery, Feinberg School of Medicine, Northwestern University, Chicago, IL 60611 USA; 10 Department of Neurology, Bioengineering, Physical Medicine & Rehabilitation, Center for Neuroengineering and Therapeutics, University of Pennsylvania, Philadelphia, PA 19104 USA; 11 Bio5 Institute and Neuroscience GIDP, University of Arizona, Tucson, AZ 85721 USA; 12 Department of Electrical and Computer Engineering, University of Arizona, Tucson, AZ 85721 USA

**Keywords:** Electrical and electronic engineering, Bionanoelectronics

## Abstract

Implantable deep brain stimulation (DBS) systems are utilized for clinical treatment of diseases such as Parkinson’s disease and chronic pain. However, long-term efficacy of DBS is limited, and chronic neuroplastic changes and associated therapeutic mechanisms are not well understood. Fundamental and mechanistic investigation, typically accomplished in small animal models, is difficult because of the need for chronic stimulators that currently require either frequent handling of test subjects to charge battery-powered systems or specialized setups to manage tethers that restrict experimental paradigms and compromise insight. To overcome these challenges, we demonstrate a fully implantable, wireless, battery-free platform that allows for chronic DBS in rodents with the capability to control stimulation parameters digitally in real time. The devices are able to provide stimulation over a wide range of frequencies with biphasic pulses and constant voltage control via low-impedance, surface-engineered platinum electrodes. The devices utilize off-the-shelf components and feature the ability to customize electrodes to enable broad utility and rapid dissemination. Efficacy of the system is demonstrated with a readout of stimulation-evoked neural activity in vivo and chronic stimulation of the medial forebrain bundle in freely moving rats to evoke characteristic head motion for over 36 days.

## Graphical abstract

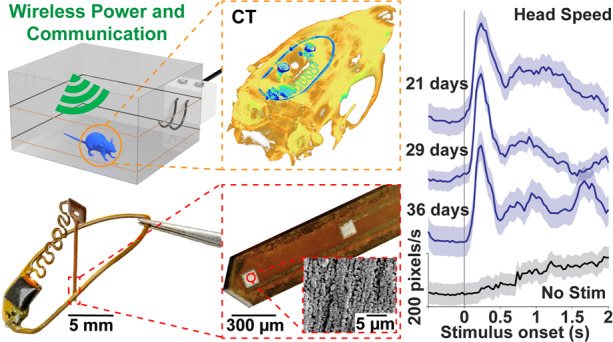

## Introduction

Wireless battery-free investigative tools for targeted neurostimulation of the brain have become important to expand neuromodulation to freely moving small animal subjects^1–5^. Continuous wireless power transfer (WPT) to the implants enables ultrathin platforms that are fully subdermally implantable, which reduces infection risk and, more importantly, removes the need for tethers to enable experiments in naturalistic environments^3^. Recent examples include the first demonstration of neuromodulation in freely flying birds^6^ and in multiple socially behaving rodents^4^, both of which would be difficult or impossible to achieve with standard tethered approaches. These current demonstrations utilize optogenetic stimulation, which is a powerful tool for exploratory research because of cell-type-specific modulation capabilities and minimal electronic hardware requirements, which enable subdermal embodiments that are scalable and feature small footprints. While optogenetics informs translational approaches^7^, direct translation is difficult because of the lack of opsin expression in human subjects.

Current clinical neuromodulation therapies, such as deep brain stimulation (DBS) for movement disorders, utilize electrical stimulation. A key challenge in DBS is identifying appropriate stimulus parameters and dosing^8^, resulting in up to 50% of DBS patients experiencing side effects^9^. The need for parameter optimization and mechanistic insight into DBS therapies motivates the demand for chronic electrical stimulation tools for small animal models such as rodents. In addition to DBS, chronic electrical stimulation is integral to emerging sensory neuroprostheses that restore sight, hearing, and the sense of touch after neurological injury or disease^10^. The full stimulus parameter space is rarely explored in neuroprosthetic studies, increasing the reliance on unnatural evoked sensations. With technologies that enable electrical stimulation in freely behaving rodent models, artificial sensory encoding paradigms could be optimized^11^.

Current battery powered and tethered methods for chronic stimulation in rodent models complicate studies. Due to the bulky nature of batteries that require frequent charging between experiments and tethered approaches requiring animal care and constant interaction with test subjects to prevent entanglement^12^, results in current techniques impacting subject behaviors^1^. A wireless, battery-free and fully implantable device would alleviate these issues. However, technological hurdles have, up to this point, prohibited such a device due to requirements for pulse timing, voltage and current modulation, and biphasic stimulation, which are not easily realized in small footprints with commercial components that enable scalable fabrication and rapid dissemination. In this work, we present devices that overcome these current technological challenges by using digitally addressable stimulators that utilize off-the-shelf components with ultrasmall footprints that leverage highly optimized antenna designs and custom one-way communication protocols to enable subdermally implantable wireless, battery-free neuromodulators with real-time voltage-controlled biphasic stimulation capabilities.

## Results

### Wireless DBS device

**Fig. 1:**
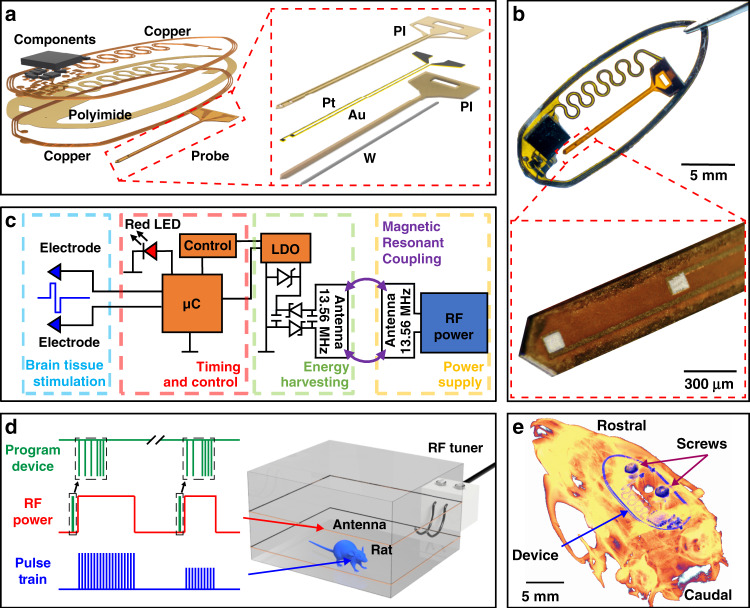
Device overview and summary of operation. **a** Exploded view illustration of the brain stimulation device (left) and electrode inset. **b** Photograph of the wireless brain stimulation device with an inset of a micrograph of the probe tip. **c** Block diagram of circuit function**. d** Mode of operation during experimental paradigms used in this work. **e** 3D µCT reconstruction of the implanted device in a rat

A monolithic design incorporates WPT capabilities in a thin flexible form factor that enables full subdermal implantation of the DBS, as shown in Fig. 1a. The flexible serpentine structure that connects the device body and the injectable stimulation probe allows for easy manipulation of the probe during surgical procedures and provides an interface that facilitates custom probe designs (Fig. 1b and Fig. S1a–c) to control impedance, depth, and spacing of the electrode^13,14^. The circuit utilizes magnetic resonant coupling at 13.56 MHz for WPT by tuning the device antenna with matching impedances to the operating frequency of the primary antenna, which in turn also operates in resonance^4,6,15–19^. The harvesting circuit uses a half-bridge rectifier with a Zener diode for overvoltage protection. An adjustable low-dropout (LDO) regulator controls the input voltage to the microcontroller (µC), which utilizes a feedback loop to adjust the stimulation voltage. Biphasic stimulation is delivered by controlling the tri-state (High, Low, High Impedance) of the μC input-output (IO) pins. Stimulation voltage is controlled by regulating the operation voltage of the digital system by changing LDO output voltage, as schematically shown in Fig. 1c. This method of biphasic stimulation allows charge balancing of the electrode, improving stimulation responses^20,21^ and minimizing tissue damage and corrosion of the electrodes during chronic stimulation^22,23^.

Stimulation parameters are wirelessly transmitted using a custom protocol that is compatible with commercially available power casting systems and features 18 bits to control amplitude, pulse width, period, and duration by modulating the radio frequency (RF) power, as shown in Fig. 1d and Fig. S2a–c. Storing these values in electrically erasable programmable read-only memory (EEPROM) allows a recall of up to 256 of these parameter spaces. The thin and flexible nature of the device allows conformal adhesion to the skull, allowing seamless recovery of the subject after implantation. The device is operational in both magnetic resonance imaging (MRI) and microcomputed tomography (µCT) systems because of careful component selection that features ferromagnetic-free materials^4,17^. This capability allows the rapid validation of probe and device placement postsurgery, as shown in Fig. 1e, as well as capabilities to expand experimental paradigms to stimulate electrically while imaging with µCT and MRI^16^.

### Electrode and stimulation control

**Fig. 2:**
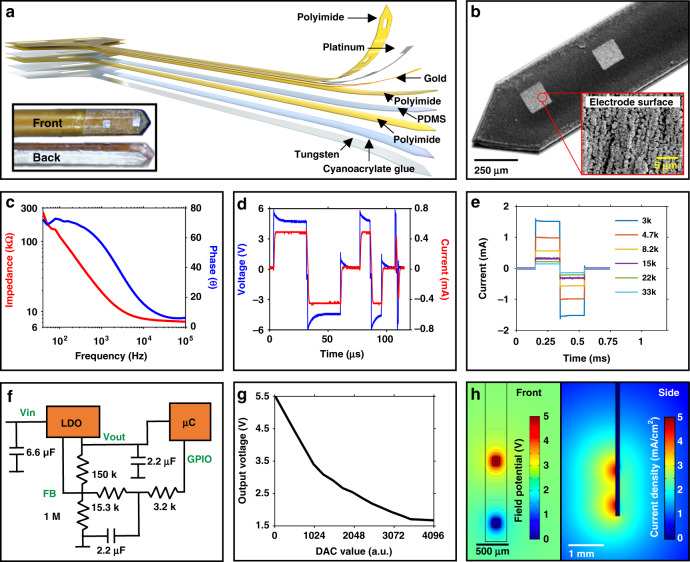
Electrode design and characterization of stimulation. **a** Layered construction of the stimulation probe with a photograph inset showing the front and back side**. b** SEM image of the probe with a magnified inset of the electrode pad**. c** Electrode impedance spectra in PBS**. d** Stimulation speed demonstration with sub-microsecond resolution. **e** Constant-voltage stimulation under varying loads**. f** Block diagram of the circuit used to control voltage**. g** Twelve-bit control over voltage used for stimulation**. h** Simulation of field potentials and current density generated at the probe tip

The probe is comprised of multiple thin layers, with a cross-sectional dimension of 220 µm × 200 µm (Fig. 2a), to minimize tissue damage during insertion while maintaining mechanical support to increase targeting accuracy, improve stimulation efficacy, and ensure repeatability of experiments^17,24,25^. Monolithic fabrication of the probe allows various electrode designs and the study of specific neural pathways^26,27^. The impedance of the probe is controlled with an engineered high surface roughness of ±400 nm (Fig. S3a) of the platinum electrode material, as shown in the scanning electron microscopy (SEM) image in Fig. 2b. Impedance characteristics of the electrode are tested before implantation to determine the voltage range needed to elicit neural activation, as shown in Fig. 2c. The direct use of µC IO enables high-frequency stimulation pulses up to 20 µs and a frequency of 50 kHz. Figure 2d shows corresponding current and voltage traces with an electrode impedance of 10 Ωk at 1 kHz. Current and voltage output is stable under a wide range of loads, as shown in Fig. 2e. To minimize footprint, an input voltage adjustment to the µC is used to control stimulation amplitudes. The resulting circuit footprint (20 mm^2^) is >10x smaller than that of other wireless electrical stimulation tools^28^ and enables designs to be adapted to a variety of animal models^4^. The voltage amplitude (1.5–5.5 V) of the biphasic stimulation is controlled through an analog feedback loop (Fig. 2f) through the adjustable LDO, which is optimized to control the µC input voltage within the voltage range of the µC supply with up to 12 bits of resolution, as shown in Fig. 2g and Fig. S3b. Finite element simulations of the field potential and current density of the electrode design with 1 mm spacing are shown in Fig. 2h. The results enable estimation of effective stimulation radius^29,30^, which, with biphasic pulses of 5 V, elicits a response up to ~2.3 mm from the electrode sites (Fig. S3c) when considering a current density stimulation threshold of >0.97 mA/cm^2^ ^31^.

### Wireless operation and mechanical properties

**Fig. 3:**
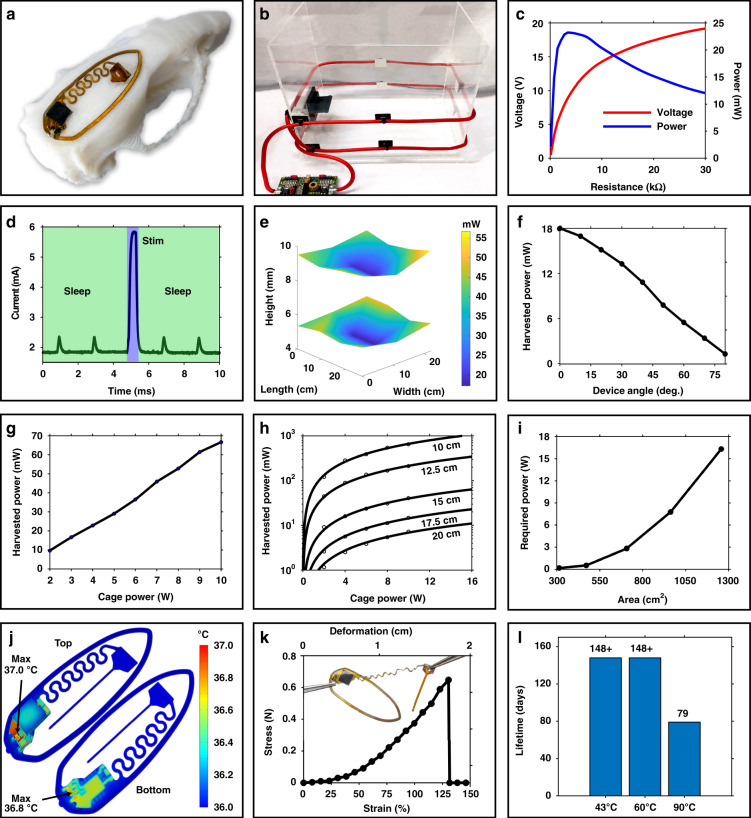
Power harvesting, thermal, and mechanical testing of the device. **a** Photograph of the device mounted on a dimensionally accurate 3D printed adult rat skull. **b** Photograph of the experimental arena used for in vivo stimulation studies. **c** Harvesting power vs. load curve in the center of the arena (22 cm × 28 cm) with RF input power of 3 W. **d** Current consumption during operation of the device during a single stimulation pulse. **e** Spatially resolved energy harvesting capability at two heights of the miniaturized device in a cage with dimensions of 22 cm × 28 cm and 3 W RF input. **f** Harvested power vs. angular misalignment between the device and cage antenna in the center of the arena (22 cm×28 cm) with RF input power of 3 W. **g** Power harvested between RF powers of 2 W and 10 W. **h** Power harvested in the center of two turn circular cages with increasing cage radius. **i** RF power needed for stable operation with a 20% safety factor in two turn circular cages with increasing cage radius. **j** Steady-state thermal impact simulation of device under continuous operation in saline. **k** Mechanical characteristics of serpentine interconnect. **l** Accelerated rate testing of device encapsulation in PBS

Figure 3a shows the dimensional optimization of the secondary antenna that allows mounting of the device on the adult rat skull while maximizing its power harvesting capabilities. The secondary antenna utilizes a 6-turn dual-layer design (70 µm trace width, 70 µm trace space) that optimizes power delivery while minimizing the secondary antenna cross-sectional dimensions (500 µm × 190 µm) to result in the smallest possible device footprint with a weight of only 46 mg, which enables faster recovery and easier implantation. For our experimental paradigm, the device is powered in a two-turn primary antenna operating at 13.56 MHz, circumferentially attached to a 28 cm × 22 cm cage at heights of 4 and 8 cm, as shown in Fig. 3b. Experimental paradigms are not limited to this antenna configuration. Characterization of harvesting capabilities of the device in several experimental arenas important for both behavioral^32^ and therapeutic^33^ studies are shown in Fig. S4a–d and indicate ample harvested power for device operation in these experimental enclosures. Commercial power casting systems with the ability to provide up to 12 W of RF power (Neurolux Inc.) and the capability to interface with additional peripheral hardware such as levers and buttons to control stimulation delivery can be used for closed-loop control during behavioral experiments that study empathy, attention, feeding, and addiction^34–37^. The secondary antenna design of the fully implantable device is optimized to harvest peak power (18 mW) at 5.5 V in the center of a 28 cm × 22 cm cage, as shown in Fig. 3c, when powered with 3 W of RF power. This harvesting ability is significantly greater than previous WPT designs that have been demonstrated with more RF input power and smaller experimental arenas^4,15,16^. The device consumes an average of 9.35 mW during stimulation epochs with a peak consumption of 29 mW for 0.6 ms during 5 V stimulation, as shown in Fig. 3d. Lower average power consumption is achieved by optimizing the µC firmware with a 1 MHz clock speed and sleep events that suspend peripheral components of the digital system. Three-dimensional mapping of power is performed at heights of 5 cm and 10 cm in a 28 cm × 22 cm cage with 3 W of RF power, as shown in Fig. 3e, indicating sufficient power throughout the experimental arena to drive stimulation. The effects of angular misalignments is also investigated in the center of the 28 cm × 22 cm cage with 3 W of RF power, as shown in Fig. 3f, indicating a linear reduction in harvesting capabilities when the device is rotated relative to the primary antenna. Harvesting capabilities during misalignment of the device position and angle with respect to the cage antenna indicates stable operation of the device during various naturalistic motions, such as rearing^38^. To accommodate these behaviors of the animal, the device requires a minimum safety margin of 1.2, which is measured relative to the lowest power availability within the cage^4^. This device provides a safety margin of ~2, enabling stable operation in a wide variety of conditions. Cage powers can be adjusted and feature a linear correlation of harvested power, as shown in Fig. 3g, measured in the center of a 28 cm × 22 cm cage. This characterization can be used to estimate the power need for larger experimental enclosures. This is demonstrated by operation in increasing cage dimensions (10–20 cm, radius) (Fig. 3h). Using a safety margin of 1.2 to provide continuous device operation, RF power requirements are calculated for various cage areas, as shown in Fig. 3i, resulting in a maximum arena size of 999 cm^2^ with 10 W of RF power.

Steady-state simulations of device heating resulting from thermal losses of active and passive components in the circuit are investigated in a phosphate-buffered saline (PBS) solution (36 °C) under natural convective heat transfer during continuous operation (>500 s). The results shown in Fig. 3j indicate a maximum increase in device temperature of <1 °C, which is within safety requirements for implanted medical devices according to the American Association of Medical Instrumentation^39^.

Figure 3k shows the mechanical characteristics of the serpentine interconnect that tethers the stimulation probe to the device body. The serpentine structure allows strains up to 125%, enabling easy articulation of the probe during implantation to target a broad range of brain regions. Device stability is investigated with an accelerated rate test, and the device lifetime was estimated using Arrhenius scaling^25^. The devices are submerged in a PBS solution and heated in an oven in a closed container to avoid evaporation. Devices subjected to 90 °C failed after 79 days. Devices tested at both 60 °C and 43 °C are still operational at the submission of this study, as shown in Figs. 3l and S5, indicating an in vivo lifetime of tens of months. Polyurethane encapsulation, which is used for some devices in this work that do not require extensive chronic stability and has the advantage of not requiring cleanroom processing with specialized tools, exhibits a lifetime of 34 days at 43 °C (Fig. S5a), which is sufficient for work with anesthetized animals and for short studies in freely moving subjects (Fig. S5b).

### Evoked neurophysiological activity

**Fig. 4:**
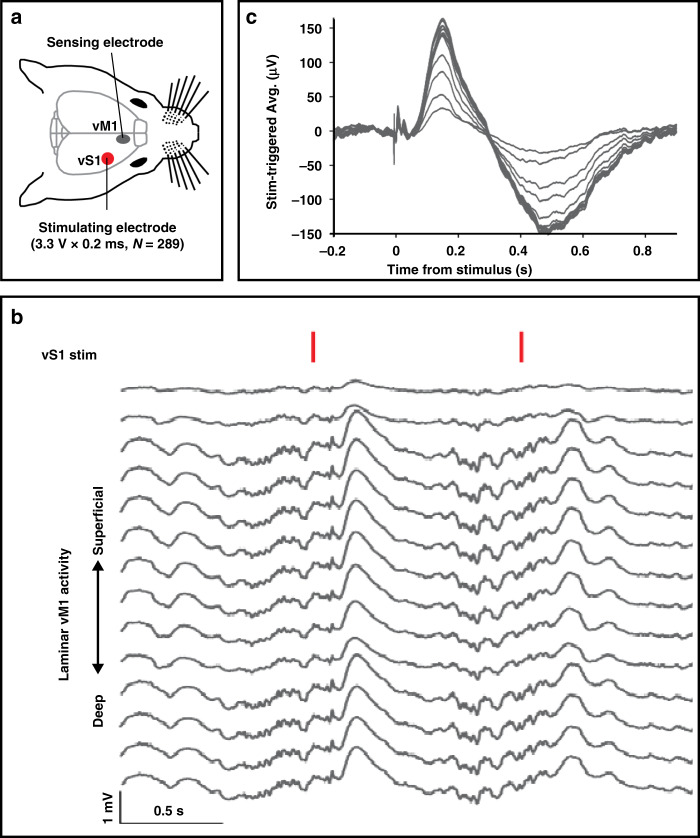
Stimulation and recording of neurological responses. **a** Illustration of acute experiment showing the location of wireless stimulation site (vibrissal primary somatosensory cortex, vS1) and wired recording site (ipsilateral vibrissal primary motor cortex, vM1) with a segment of vM1 recording during 1-Hz vS1 wireless stimulation (indicated by red vertical lines). **b** Segment of vM1 recording during 1-Hz vS1 wireless stimulation (indicated by red vertical lines). **c** Stimulus-triggered average of neural activity recorded on each of the vM1 laminar probe channels. In both graphs b and c, the two most superficial recordings from the 16-channel array were omitted

The efficacy of wireless, battery-free, and fully implantable stimulators is demonstrated in an acute experiment to document neurophysiological effects (Fig. S6a). In an anesthetized rat, the bipolar wireless stimulating electrode is stereotaxically placed (Fig. S6b) in the vibrissal primary somatosensory cortex (vS1). A wired recording electrode array (16-channel laminar probe) records activity in the vibrissal primary motor cortex (vM1), to which vS1 is synaptically connected (Fig. 4a). Single biphasic stimulus pulses (3.3 V × 0.2 ms, *N* = 289) are delivered at 1 Hz to vS1 as the response in vM1 is recorded (Fig. 4b). A robust stimulus-evoked potential is recorded in vM1, consisting of both early (5 ms peak) and late (150 ms peak) components that vary across cortical depth (Fig. 4c), demonstrating that wirelessly delivered stimulus pulses are capable of activating cortical circuits.

### Chronic behavioral effects

**Fig. 5:**
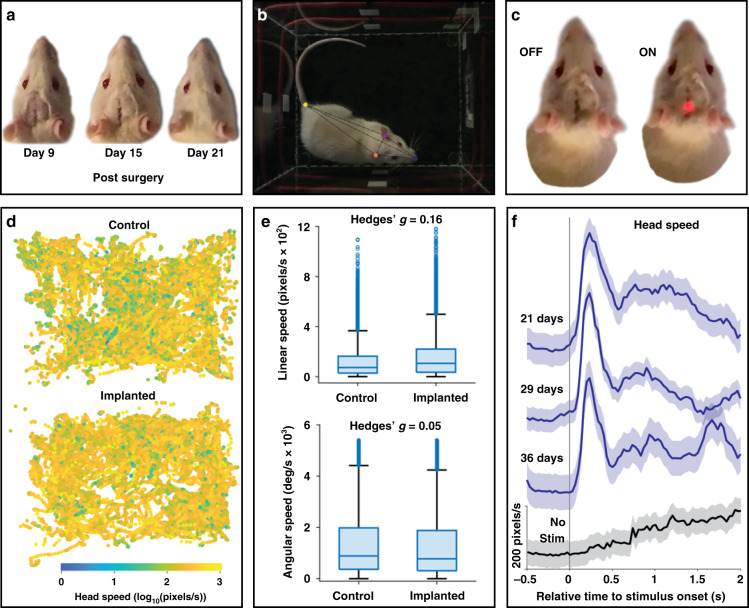
Recovery and behavior of freely moving animals. **a** Images documenting healing of the scalp over the implant. **b** Example image from overhead video used to quantify the rat’s response to wireless stimulation of the MFB. Superimposed colored circles show the pose estimates of the deep learning model used to track movement for the nose, ears, and tail base. **c** Implanted device LED visible through the scalp on a video frame immediately before stimulation (left) and at stimulation onset (right). **d** Tracked head position within the open field for a control rat and the implanted rat (from 4 × 10 min sessions each). Colors of datapoints at each position indicate head speed. **e** Distribution of linear (top) and angular (bottom) head speeds for the control and implanted rats. **f** Mean head speed (±95% CIM) relative to stimulus onset in three testing sessions (blue traces) and relative to random times when animal was at rest in a no-stimulation control session (gray trace)

Efficacy in freely behaving subjects is demonstrated in chronically implanted rats. The stimulation electrode is stereotaxically placed in the medial forebrain bundle (MFB), the wireless device body is implanted under the scalp, and subsequent sutures close the skin over the device. There are no signs of infection, irritation, or behavioral changes 1 week after implantation. The scalp completely healed over the implant within 3 weeks (Fig. 5a). In this experimental paradigm, the stimulation probe is designed to target the MFB at the level of the hypothalamus, which contains major dopaminergic pathways. MFB stimulation can activate mesolimbic dopaminergic fibers, producing a pleasing and motivating sensation^40^. MFB stimulation can also evoke forward locomotion and turning behaviors—the effect sought in this work—putatively due to activating nigrostriatal dopaminergic fibers^41–43^. Stimuli are manually triggered during sessions in which the rat explores an open field (28 cm × 22 cm). From overhead video, motion of the rat is quantified using a deep learning model (Fig. 5b). Stimulus timing is validated by the illumination of a red LED on the device under the scalp (Fig. 5c), indicating to the operator that the device is active. In addition to scalp healing, locomotor abilities are not impacted by the implant. The impact of the device is measured by tracking exploration in an open field in a manner similar to that of a naïve control rat (Fig. 5d). The effect sizes of differences in linear and angular head speed between the control and implanted rats are small: Hedges’ *g* = 0.16 and *g* = 0.05, respectively (Fig. 5e). Finally, wireless nigrostriatal stimulation is effective in reliably evoking rapid forward movement (Supplementary Video 1). The evoked movement is tightly locked to stimulus onset, with a mean latency to peak speed of 233 ms. Movement is consistently evoked for over a month following implantation (Fig. 5f). The device eventually failed by postimplant day 44, possibly due to encapsulation failure^44^ or erosion^45^ of the electrode interface. Both are typical failure mechanisms of implantable electrical stimulation devices and are active fields of research.

## Discussion

The flexible wireless DBS device introduced here is capable of delivering controlled charge-balanced stimulation pulses that can be regulated digitally in their delivery frequency (down to 20 µs pulses with up to 25 kHz frequency) and voltage amplitude (1.5–5.5 V). The system can be used in a broad range of chronic experimental paradigms with freely moving subjects in ethologically relevant naturalistic environments, which cannot easily be achieved with battery-powered or tethered devices. By using a wireless, battery-free approach, we minimize the effect on subject mobility (device weight 46 mg) and risks of infection or injuries that arise from group housing. Optimization of the device dimensions facilitates easy subdermal implantation, allowing fast recovery times and no change in animal behavior compared to naïve control animals. The technologies presented here further enable a wide range of customizations and the adoption of various electrode designs, such as those presented here or other flexible electrode technologies, that can be exchanged to target brain regions of interest. In addition, real-time wireless control over stimulation parameters in cage dimensions of up to 999 cm^2^ enables a wide range of experimental paradigms. The small size and minimal footprint of the implants and the use of ferromagnetic-free off-the-shelf components enable compatibility with noninvasive 3D imaging of subjects and facilitate the possibility of broad dissemination with existing scaled manufacturing technologies. Successful chronic experiments and scalable technologies demonstrated in this work suggest potential for widespread adoption in neuroscience research and will enable future studies to explore chronic electrode performance, stimulation-induced neural plasticity, and chronic closed-loop behavioral studies in freely moving subjects. The technology may also serve as a platform to enable wireless and battery-free operation for features such as neural recording and muscle interfaces^46^.

## Materials and methods

### Flexible circuit fabrication

Copper traces were defined on Pyralux (AP8535R; constituent layers: 17.5-µm copper, 75-µm polyimide, and 17.5-µm copper) using a UV (355-nm) laser ablation system (LPKF; Protolaser U4). The flexible circuits were cleaned in stainless steel flux (Superior Flux and Manufacturing Company; Superior #71) for 2 minin an ultrasonic cleaner (Vevor; Commercial Ultrasonic Cleaner 2 L) and rinsed with deionized (DI) water. Via connections were established manually with copper wire (25 µm) and low-temperature solder (Chip Quik; TS391LT). Combinations of 0201 capacitors (108 pF) were used to tune the power harvesting antenna. A half-bridge rectifier was built with low-capacitance Schottky diodes (Skyworks) and three 0201 capacitors (2.2 µF). A Zener diode (Comchip Technology Corporation; 5.6 V) provided overvoltage protection to limit the supply voltage to the adjustable LDO (Maxim Integrated, MAX38902C) used to stabilize the input voltage to the µC (Atmel; ATtiny 84 A). A custom programmer board using Arduino as ISP was used to program the µC before mounting on the circuit. The µC provided control over visual indication through a red LED with a 3.3 kΩ current limiting resistor, controlled timing of electrical stimulation, and LDO output voltage. The components were reflowed with a hot air gun at 350 °C using a low-temperature solder (Chip Quik; TS391LT). The devices were tested with a reflection bridge (Siglent; SSA 3032X; RB3X20), and additional tuning capacitors were added to provide the lowest-voltage standing wave ratio at 13.56 MHz. Silver particle-filled epoxy (Model 8331, MG Chemicals Inc.) was used to establish an electrical connection to the probe and cured at 65 °C for 30 min. The connection was mechanically joined using UV curable glue (Damn Good; 20910DGFL) and cured under a UV lamp (24 W, 10 min). A tungsten foil (Alfa Aesar, CAS# 7440-33-7) was defined through laser ablation (LPKF; Protolaser U4) and mounted to the back of the probe using cyanoacrylate as a structural support.”

### Stimulation electrode fabrication

Figure S7 presents a schematic illustration of the fabrication and layout of the stimulation electrode. The first step involves the removal of the copper layer from a flexible copper-clad polyimide film (AP9111R, DuPont), yielding a 25 µm thick polyimide with a surface roughness of ±400 nm. This substrate served as a template to introduce the surface roughness on subsequent thin-film metal layers. A photolithography and lift-off process, followed by electron beam evaporation (Cr/Au/Ti/Pt, 5/100/5/100 nm), defined both the stimulating metal electrode and the interconnects. A coating of polyimide (PI2545, HD MicroSystems; 2 µm in thickness) served as the passivation layer, opened the contact pad by reactive ion etching (O2, 100 mTorr, 100 W, 20 sccm, 10 min) and exposed the platinum (area of 130 µm × 130 µm) stimulation electrode. A 75 µm thick polyimide film served as the supporting substrate for short electrodes (below 4 mm), and the addition of a tungsten shuttle (50 µm thickness) served as a stiffener to enable high-accuracy targeting. Both supporting substrates were attached to the electrode film with an adhesive layer (polydimethylsiloxane). The laser cutting process (ProtoLaser U4, LPKF) completed the formation of the electrode layout.

### Electrode characterization

The electrical behavior of the microelectrodes was studied in Dulbecco’s PBS solution (14190-136, Gibco, Life Technologies). A three-electrode electrochemical cell including a stimulating electrode, Pt wire, and Ag/AgCl electrode (MF-2052, BAS) as the working, counter, and reference electrodes, respectively, was immersed in PBS. Electrochemical impedance spectroscopy (Autolab PGSTAT128N) was used to measure the impedance of the working electrode at frequencies ranging from 0.1 Hz to 100 kHz under an applied voltage input of 5 mV. The stimulation output of the wireless, battery-free device was characterized by programming commands through RF modulation controlled by an Arduino Nano (Atmel; ATmega328) that communicates over a serial port to a computer. The devices were wirelessly programmed through RF modulation with 3 pulse widths with an amplitude of 5 V. The current and voltage was recorded with a source measure unit (Keithley, Model 2450 SourceMeter®) with a 10 kΩ load. The device with 5 V stimulation and the current was measured (LowPowerLab; CurrentRanger) with various resistive loads. The amplitude of stimulation was wirelessly programmed and measured with an oscilloscope (Siglent; SDS 1202X-E) with a 10 kΩ load.

### Encapsulation

The devices were rinsed with IPA for 10 min and air-dried. UV curable glue (Damn Good; 20910DGFL) was added over the components, then cured under a UV lamp (24 W, 10 min) and degassed in an oven (100 °C, 5 min) to add mechanical protection to the solder interface of the surface-mounted components. The conformal coating was achieved by Parylene-C encapsulation: the electrode tip was covered with polyimide sheets on either side of the probe to protect the electrode surface from the coating, and the seams were held together using parafilm. The devices were suspended along a wire and encapsulated using the Parylene P6 coating system (Diener electronic GmbH, Germany) with 2 coating runs each using 5.0 g of Parylene-C dimer for a total thickness of ~18 µm, covering the entire device surface conformally with Parylene-C to provide a moisture barrier and a biocompatible biointerface. The encapsulation thickness was calculated from measurements using a profilometer (Tencor P15, KLA) and subsequently controlled using Parylene-C dimer weights (Fig. S8). Polyurethane-coated devices were obtained by covering the probe tips, hanging the devices on a wire, spraying them with premium polyurethane conformal coating (4223 F, MG Chemicals Inc.), and curing at 90 °C for at least 12 h. The devices were finally dip coated with PDMS (SYLGARD™ 184 Silicone Elastomer kit) and cured at 80 °C for 10 min after the excess was removed using a syringe.

### Power harvesting characteristics

Voltage and power harvesting characteristics were collected (Aneng; AN8008) using a load resistor in the center of a 28 cm × 22 cm arena powered with a dual loop antenna with 3 W of RF power. A shunt resistor (1.6 kΩ) was used to match the system load during harvested power measurements in 3D maps at heights of 6 and 10 cm and with angular misalignment between the cage antenna and device antenna using an oscilloscope (Siglent; SDS 1202X-E) in a 28 cm × 22 cm arena with 3 W of RF power. The harvested power was measured (Siglent; SDS 1202X-E) in the center of the cage with RF power ranging from 2 to 10 W. Current consumption was recorded with a modified current meter (LowPowerLab; CurrentRanger) and acquired using an oscilloscope (Siglent; SDS 1202X-E). Harvested power was measured (Siglent; SDS 1202X-E) using a shunt resistor (1.6 kΩ) in the center of a dual loop antenna with diameters of 10 to 20 cm with varying powers and linearly fit between powers of 0 to 16 W. The average power consumption combined with a safety factor of 20% was used to determine the required delivered RF power for stable operation in the centers of varying sizes of circular cages.

### Electrical and thermal simulation

Finite-element simulation of field potentials and current density was performed in COMSOL ® Multiphysics with a stimulation of 5 V. Accurate probe dimensions and material properties for dielectric, electrical conductivity, and relative permittivity was used for each material, polyimide (6.66 S/m, 3), platinum (9.4e6 S/m, 0.0039), and saline (1.3 S/m, 75), and analyzed during the first biphasic stimulation pulse.

Finite-element simulation of heat transfer in solids and fluids after 1000 s of continuous operation of the brain stimulation device was performed in COMSOL ® Multiphysics. The integrated components, copper traces, and polyimide were accurately modeled, and natural convection in saline with an initial temperature of 36 °C to mimic average temperature in rats was used as starting parameters. The heating power applied to the components was as follows: μC 8.6 mW; LDO 5 mW; rectifier 10 mW; LED 0.5 mW; LED resistor 0.5 mW. The thermal conductivity, heat capacity, and density of different materials were as follows: component mold compound (0.5 W m^−1^ K^−1^, 1000 J kg^−1^ K^−1^, and 1350 kg m^−3^), inner dies (130 W m^−1^ K^−1^, 678 J kg^−1^ K^−1^, and 2320 kg m^−3^), copper (400 W m^−1^ K^−1^, 385 J kg^−1^ K^−1^, and 8900 kg m^−3^), polyimide (0.2 W m^−1^ K^−1^, 1100 J kg^−1^ K^−1^, and 1470 kg m^−1^), and saline (0.6 W m^−1^ K^−1^, 4180 J kg^−1^ K−1, and 1000 kg m^−3^).

### Mechanical testing

The device was mounted on a scale (Mettler Toledo; AB104-S). The probe was fixed to a custom 3D printed slider to measure the displacement of the device as it was stretched using an electronic digital caliper. The serpentine structure was stretched in 1 mm increments.

### Encapsulation testing

Three encapsulated devices (PDMS, polyurethane+PDMS, and Parylene-C+PDMS) were submerged in 0.01 M phosphate buffer, 0.0027 M potassium chloride and 0.137 M sodium chloride (Sigma, P4417) in sealed glass vials at 43 °C and recorded daily to visually check the operation using an indicator LED. This test was also conducted with devices coated with Parylene-C and PDMS in PBS temperatures of 60 °C and 90 °C and checked daily for device operation.

### Acute in vivo testing

Both the acute and chronic experiments were approved by the Institutional Animal Care and Use Committee of the University of Pennsylvania. The study used three male Sprague-Dawley rats (Crl:SD, 275–325 g): one for the acute experiment, one chronically implanted, and one unimplanted control. For the acute experiment, the rat was anesthetized with intraperitoneal injection of ketamine (60 mg/kg) and dexmedetomidine (0.25 mg/kg) and placed in a stereotaxic frame. Throughout the procedure, depth of anesthesia was monitored by respiratory rate and pedal reflex and maintained at a surgical plane with additional injections of ketamine as needed. A large craniotomy was performed to expose the right vS1 and vM1. A 16-channel laminar probe with 50 µm electrode diameters (Microprobes for Life Science) was placed in vM1 to record stimulus-evoked activity. The recordings were referenced to a remotely placed subdural wire, with a 00-90 skull screw placed in the frontal bone to serve as the ground. The wireless stimulating electrode was placed in vS1. Single electrical pulses (3.3 V × 0.2 ms) were delivered to vS1 at 1 Hz while recording from the vM1 array using an Intan RHS system (Intan Technologies).

### Chronic implantation

The rat was anesthetized with 5% isoflurane in oxygen and placed in a stereotaxic frame. Buprenorphine SR (1.2 mg/kg) was administered subcutaneously for long-acting analgesia. Throughout the procedure, depth of anesthesia was monitored by respiratory rate and pedal reflex and maintained at a surgical plane with 1.5–2.5% isoflurane. A 1.5-mm diameter craniotomy was centered on a point 2.8 mm posterior to bregma and 1.7 mm lateral to midline. A small slit was made in the dura. Two 00-90 skull screws were implanted anterior and posterior to the craniotomy. The device was mounted on a manual micromanipulator using a toothless micro alligator clip attached to the base of the probe. The tip of the probe was slowly lowered to a depth of 8 mm below the dura into the MFB at the level of the lateral hypothalamus^40^. While lowering, the device antenna was tucked underneath the scalp. After lowering, a small amount of acrylic dental cement was applied to bond the probe to the skull screws. After removing the clip, more acrylic was applied to cover the probe and screws entirely while ensuring that no acrylic came into contact with the antenna or other parts of the device. The scalp was then sutured over the implant. After one week of recovery, the sutures were removed, and the experiments began.

### Behavioral experiments and analysis

Experimental sessions consisted of placing the chronically implanted rat or the control rat in the wireless stimulating open field and using videography to capture the animal’s pose during exploration. In the case of the implanted rat, stimulation was occasionally triggered manually by a battery-powered switch. To avoid an acoustic startle response, care was taken to ensure that there was no auditory indication of the electrical stimulus. Stimulation was delivered during periods of immobility to more easily distinguish stimulus-evoked movement from volitional movement. Overhead video was recorded in 1080p HD at 30 frames/s. Data analysis began by extracting pose and stimulus timing information from the videos. Pose estimation was performed using DeepLabCut^47^. A convolutional neural network was trained on a GPU-accelerated virtual machine in Google Colaboratory to track the position of the nose, ears, and tail base. Stimulus onset was determined from the illumination status of the implanted LED on each frame using an image processing script in MATLAB. Then, the speed of each tracked body part was estimated using the finite difference approximation after removing pose estimates with likelihood scores <0.995. Finally, the mean evoked speed across stimuli and 95% confidence intervals on the mean (CIM) were computed in a 2.5-s window around stimulus onset.

### µCT Imaging

At the conclusion of the chronic experiment, the rat was euthanized with an intraperitoneal injection of sodium pentobarbital and transcardially perfused with heparinized saline followed by 10% neutral buffered formalin (NBF). The head was soaked in NBF for 24 h before removing the soft tissues around the skull and loading it in a 50 ml centrifuge tube for imaging. The whole head was scanned using 90 kVp with a copper filter at 14.6 μm isotropic resolution (μCT45 scanner, Scanco Medical). The µCT DICOM stack was imported into ImageJ, and all dimensions were deduced to 25% of its original size. Image stack contrast and brightness were automatically adjusted to the background. The plugin volume viewer was used to view the CT image as a volume with a 2D gradient thermal LUT color overlay.

## Supplementary information

Supplementary figure
