# Supplementary material for: Wireless, battery-free, and fully implantable electrical neurostimulation in freely moving rodents
Source: Microsyst Nanoeng. 2021 Aug 13;7:62. doi: 10.1038/s41378-021-00294-7 (PMC8433476; doi:10.1038/s41378-021-00294-7)
Supplement: Supplementary file 1 — Supplementary figure [file 41378_2021_294_MOESM1_ESM.pdf]

Supplementary Information

Wireless, battery free and fully implantable electrical neurostimulation in freely moving rodents

Alex Burton, Sang Min Won, Arian Kolahi Sohrabi, Tucker Stuart, Amir Amirhossein, Jong Uk Kim, Yoonseok Park, Andrew Gabros, John A. Rogers, Flavia Vitale, Andrew G. Richardson, Philipp Gutruf

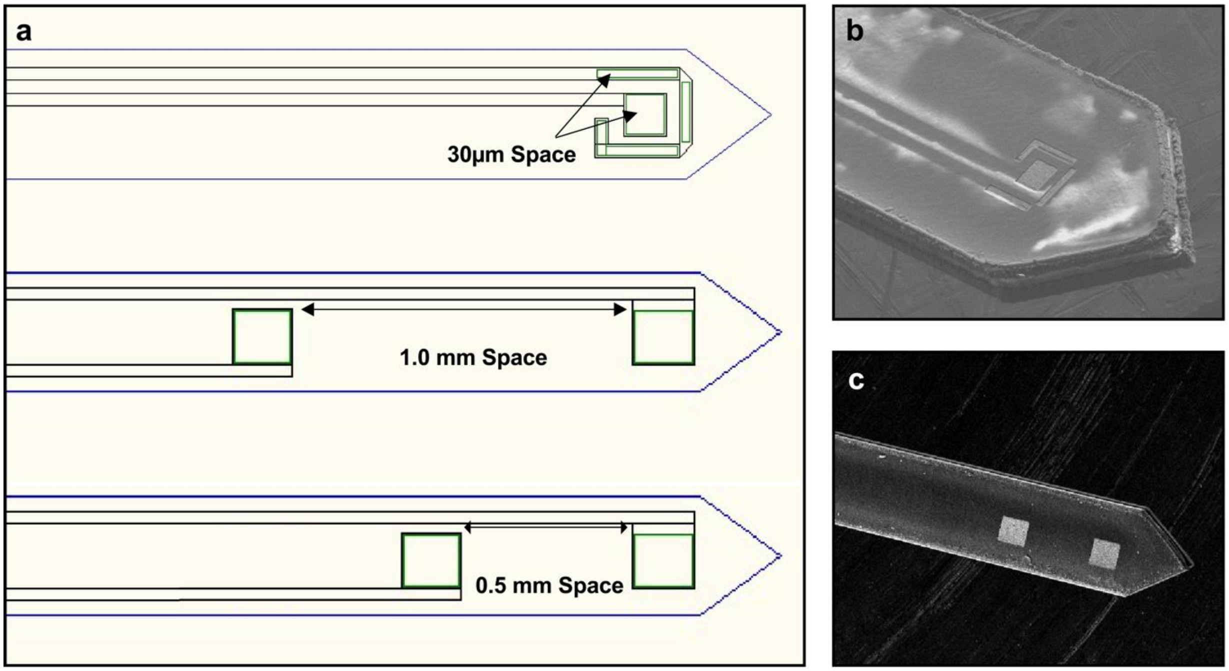

Figure S1. **a** Three electrode designs with various dimensions of electrode placement and patterns. **b** SEM image of the electrode tip (design 1). **c** SEM image of the electrode tip (design 2).

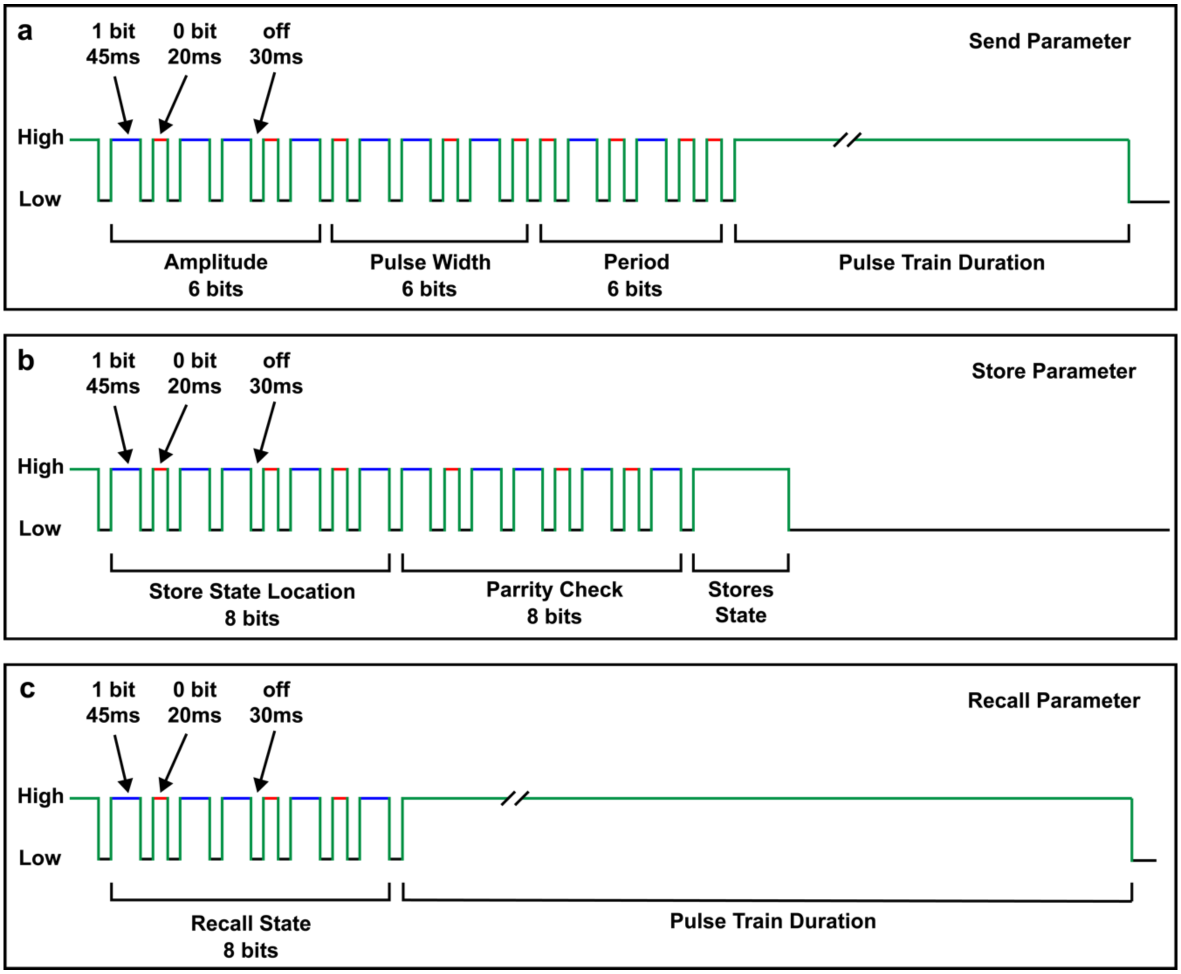

Figure S2. **a** Device communication protocol to control pulse train parameters. **b** Device communication to store current pulse train parameters. **c** Device communication to recall stored pulse train parameter.

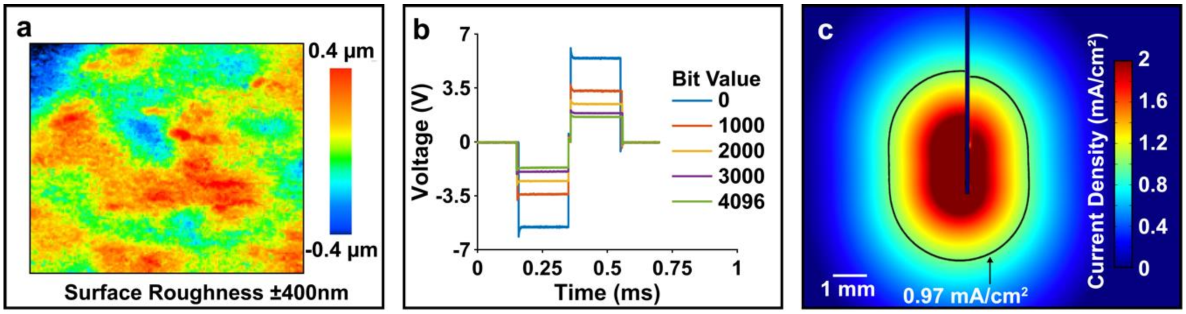

Figure S3. **a** Optical profilometer image of surface roughness of the electrode surface. **b** Time base amplitude control of biphasic stimulation measured over a 10 KΩ load. **c** Current density simulation showing effective stimulation range.

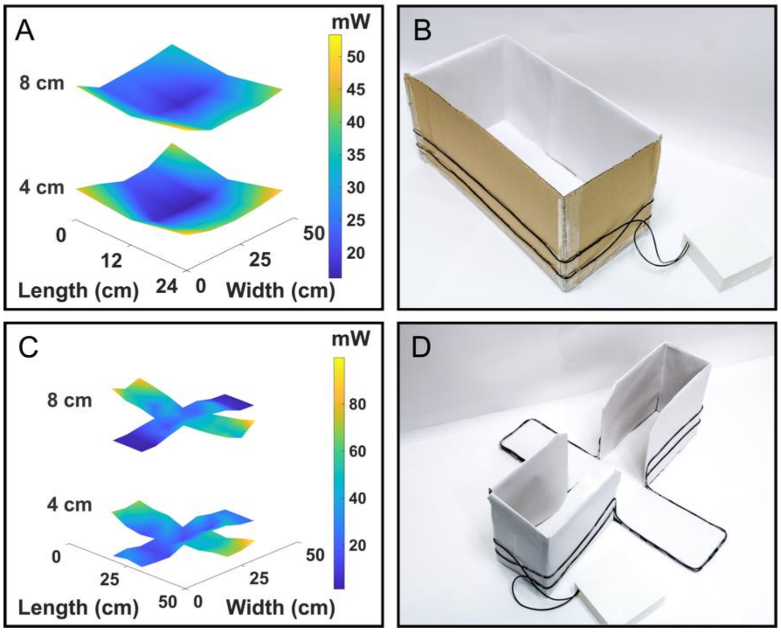

Figure S4. **a** Three-dimensional map of power harvested in a 24 cm x 50 cm cage at heights of 4 cm and 8 cm with 5W of RF power. **b** Photo of the 24 cm x 50 cm cage used to measure power harvesting. **c** Three-dimensional map of power harvested in a 50 cm x 50 cm plus maze with 10 cm wide arms at heights of 4 cm and 8 cm with 5W of RF power. **d** Photo of the 50 cm x 50 cm plus maze used to measure power harvesting.

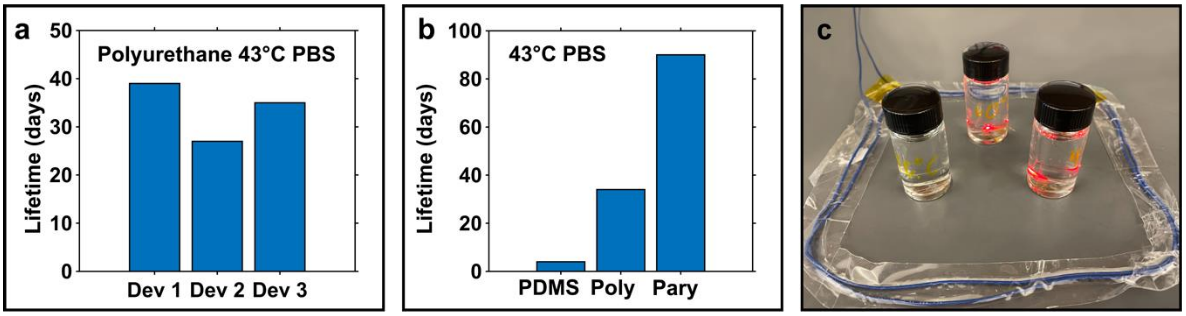

Figure S5. **a** Lifetime of polyurethane coated devices in 43 °C PBS solution. **b** Lifetime of PDMS vs PDMS and polyurethane vs PDMS and Parylene-C coated devices in 43 °C PBS solution. **c** Photographic image of the device under test.

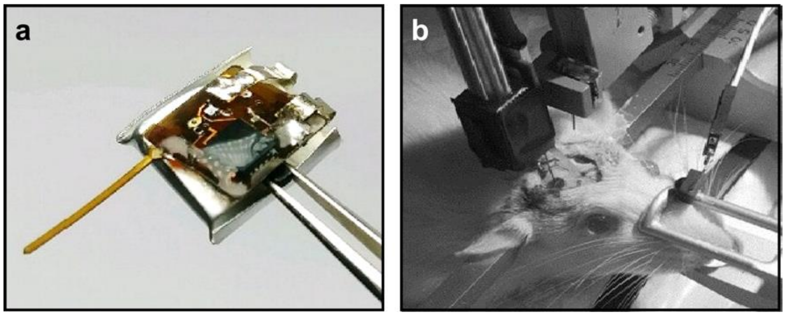

Figure S6. **a** Wireless battery powered device. **b** Stereotactic alignment of the electrode probe.

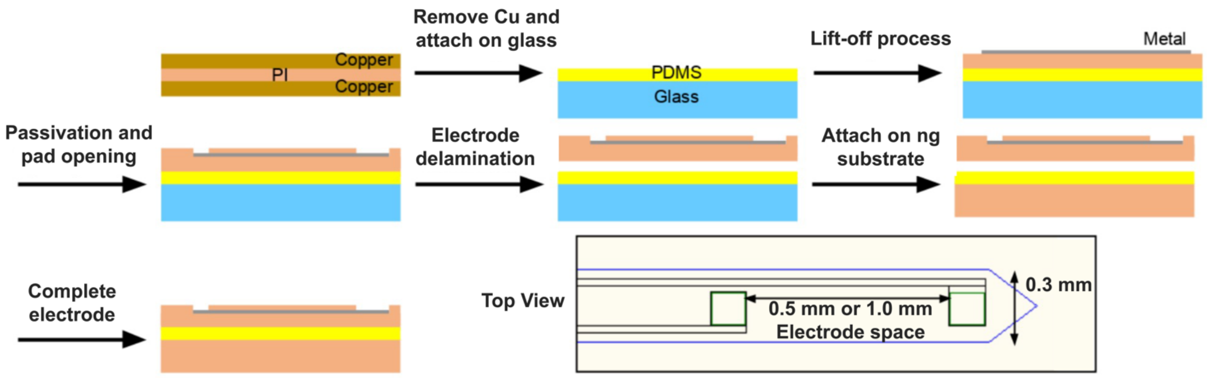

Figure S7. Microfabrication process used to make probes for deep brain stimulation.

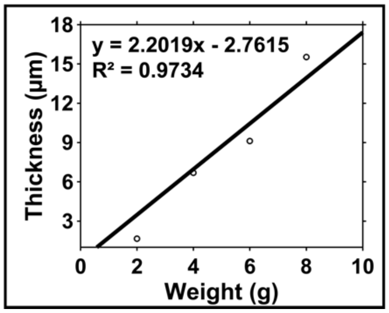

Figure S8. Coating thickness based on weight of Parylene-C dimer used.
